# A Case of Clear Cell Hidradenoma Found During Abdominal Cyst Excision

**DOI:** 10.7759/cureus.22957

**Published:** 2022-03-08

**Authors:** Niritta Patel, Adam Kramer, Xiaoming Sun

**Affiliations:** 1 Surgery, Kansas City University of Medicine and Biosciences, Kansas City, USA; 2 General Surgery, St. Mary's Medical Center, Blue Springs, USA; 3 Dermatopathology, Quest Diagnostics, Kansas City, USA

**Keywords:** abdominal cystic mass, apical sweat glands, surgical excision, clear cell hidradenoma, abdominal cyst

## Abstract

Clear cell hidradenoma (CCH), a rare yet benign finding, is a tumor that originates from sweat glands. It mainly presents as a slow-growing cystic nodule on the scalp or trunk. We report a case of a 60-year-old man who presented with an abdominal subcutaneous mass. This mass was excised and pathology confirmed the presence of CCH, with cytology negative for malignant cells. Although benign, CCHs cannot be confirmed as such until excision and pathologic analysis, which is the standard treatment of choice.

## Introduction

Clear cell hidradenoma (CCH) is a rare benign tumor originating from apical sweat glands. It can display either apocrine or eccrine differentiation [1]. General presentation is that of cystic nodules, 5-30 mm in size, most commonly found on the scalp or trunk in women, though they may also present on the limbs and can occur in all ages [1,2]. Lesions are solitary and characterized by slow growth with potential serous discharge. Although they are benign, CCHs have a 10% rate of recurrence following surgical incision [2]. Here, we report the incidental case of CCH found in a cyst excised from the abdominal wall of a 60-year-old male. This patient initially was thought to have an epidermal inclusion cyst which was to be removed during an in-office procedure done under local anesthesia. The specimen was sent for pathologic examination that revealed CCH. We did a literature review as this was a rare and interesting finding.

## Case presentation

The patient was a 60-year-old male who underwent abdominal cyst excision. The cyst was examined during his clinic visit and was noted to measure about 1.5 cm long. His past medical history was notable for diabetes and prosthetic right leg. He had no other complaints at the time of the visit. Excision of the cyst was performed in the clinic. Following injection of local anesthetic at the incision site, an elliptical incision was made and the cyst was removed, after which skin was closed. The patient tolerated the procedure well, and postoperative course was uneventful. The incision was well approximated and well healing at the one-week follow-up, and the patient reported minimal pain.

**Figure 1:**
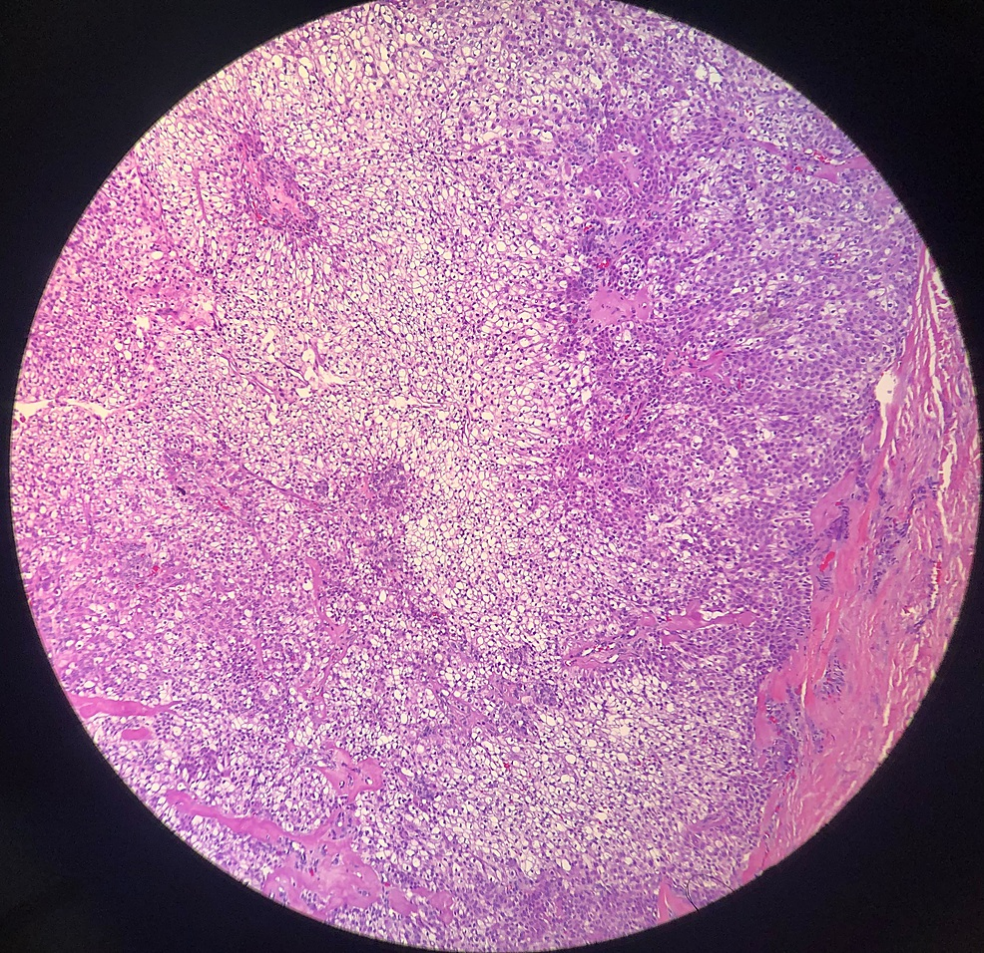
Clear cell hidradenoma consisting of squamous neoplasm with clear cell features, with no atypia or necrosis H&E staining method was used, and the image was taken at 10X magnification.

**Figure 2:**
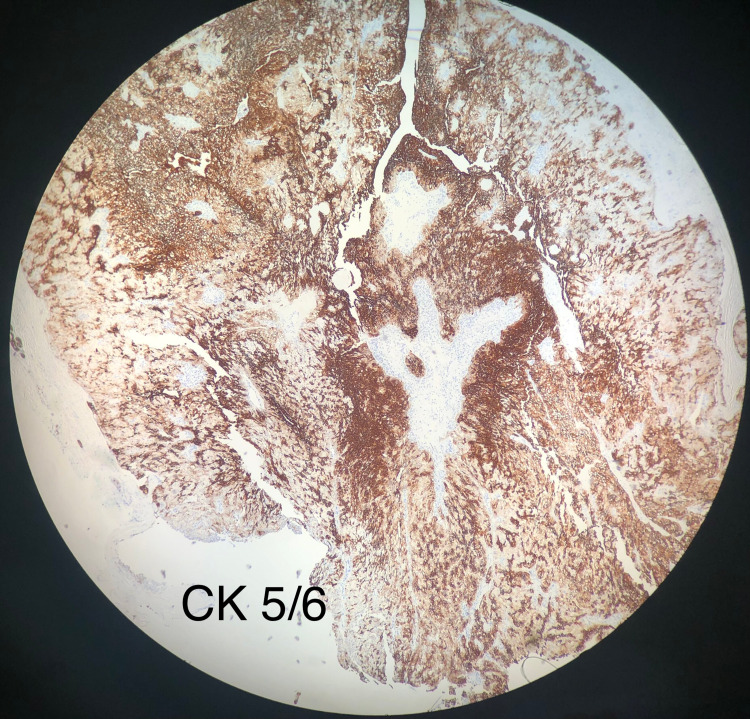
Clear cell hidradenoma staining positive with cytokeratin as shown by an increased uptake The image was taken at 10X magnification.

**Figure 3:**
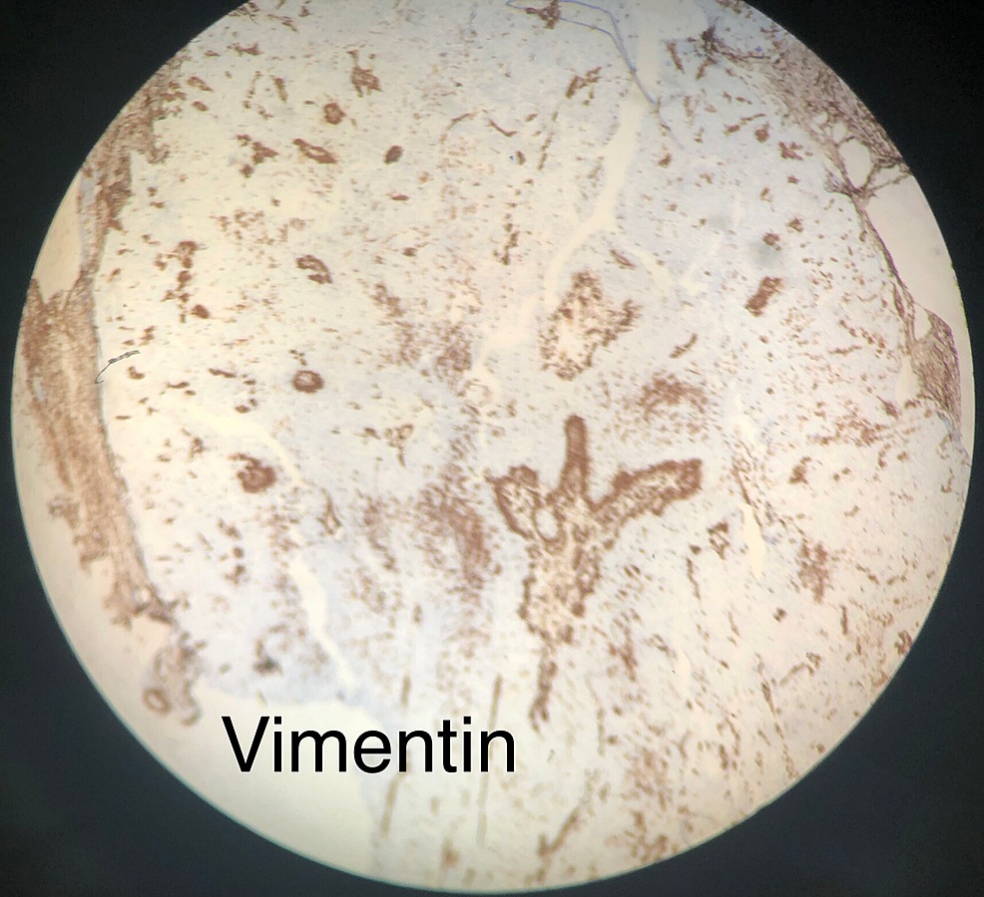
Clear cell hidradenoma staining negative with vimentin as shown by a decreased uptake The image was taken at 10X magnification.

The pathologic evaluation of the specimen revealed skin with a cystic structure elliptical in shape and yellow-white in color. Cyst contents included a granular material consisting of squamous neoplasm with clear cell features, and cells showed no significant atypia or necrosis (Figure 1). The cytology specimen was negative for malignancy. Additionally, the tumor was found to stain positive with cytokeratin (Figure 2) and negative with vimentin (Figure 3). Staining for cytokeratin and vimentin was done to rule out the remote possibility of metastases from clear cell renal cell carcinoma.

## Discussion

CCH is a benign adnexal tumor that most commonly presents on the scalp and trunk, though it also infrequently arises on the upper and lower limbs [3]. It typically manifests as a solitary, slow-growing and firm nodule ranging from 5 to 30 mm in size. Histologically, it dwells within the dermal and subcutaneous fat layers, and is covered by a layer of normal epidermis [4]. The transformation to its malignant counterpart, hidradenocarcinoma, is rare [2].

CCHs have also been recounted in case reports in which the lesion was found on locations such as the breast and hand. Mammography and ultrasonography were used to detect a 3-cm CCH in the left axillary tail of a patient [4]. The mass presented as a well-circumscribed, high-density mass on mammography and a well-defined cystic mass with hypoechogenicity on ultrasound. A distinguishing feature of CCH is its appearance as a well-circumscribed, subcutaneous, cystic or solid mass with low-to-intermediate signal intensity on T1 weighted MRI and an intermediate-to-high signal intensity on T2 weighted MRI [4]. Sonography was also used to classify a 1.5-cm mass in the subcutaneous tissue of a patient’s hand as CCH [2]. This mass gradually increased in size, leading to excisional biopsy to make a definitive diagnosis and surgical excision to relieve the patient’s discomfort. During surgical excision, the mass was found to be adhered to the dermal layer with epidermal connection, and upon removal it did not appear to invade the surrounding tissues.

Though extremely rare, CCH has also been detected among infants and young children, and should be considered among this population within the differential diagnosis of subcutaneous lesions. In one such case, a five-year-old boy was found to have a skin tumor on the left forearm that slowly enlarged over the course of six months [5]. Histopathologic examination revealed a well-defined, solid tumor with clear cell features, with no adhesion to deeper tissues. No signs of recurrence were noted following excision. In another case, CCH presented as a brownish-red nodule on the external right leg of an 18-month-old girl. The nodule had central ulceration with drainage of serous material and had gradually increased in size over a year [6]. Histopathologic examination revealed a well-circumscribed tumor containing large cells with pale eosinophilic cytoplasm, consistent with clear cell hidradenoma. Following complete excision, no signs of recurrence were noted through 13 years of follow-up. It is imperative to recognize this diagnosis as it may present differently at various ages, and follow-up is needed to track potential recurrence.

While CCH is benign, there have been cases in which CCH has involved the lymphatic system, causing it to be labeled as a tumor of uncertain malignant potential. In one such case, a 36-year-old woman presented with a subcutaneous mass in her left groin eight years after treatment for CCH in this region [7]. Tumor cells demonstrated abundant clear cytoplasm and squamoid features, with the absence of an infiltrative growth pattern by way of atypia or necrosis. These findings correlated with those of the patient’s CCH treated eight years ago, suggesting that the tumor had gained access to the lymphatic system but remained benign. This case prompted the usage of the term “tumor of uncertain malignant potential” for diagnosis, as malignant features were not noted even though the tumor appeared to have undergone lymphatic spread. In another such case study, three cases of CCH with lymph node involvement were reported, none of which had systemic involvement [8]. While all three cases displayed the involvement of inguinal lymph nodes, none of the resected lesions exhibited features of malignancy, specifically without atypia or necrosis. It is important to recognize such a diagnosis and to monitor CCH with regular long-term follow-ups to detect such a presentation, as it may initially appear to be malignant but on closer inspection may be found to be benign.

The differential diagnosis for abdominal subcutaneous masses is broad, including abscesses, sebaceous cysts, lipomas and epidermal inclusion cysts [9]. Imaging studies such as MRI can be used to detect CCH [9,10]. Treatment of choice is wide surgical excision with examination of pathology following excision to confirm the diagnosis, as done in our case. Prognosis is thought to be excellent with surgical excision, though a long-term follow-up may be needed to track potential benign metastases or malignant transformation to hidradenocarcinoma. Should transformation to hidradenocarcinoma occur, the prognosis is poor due to high rates of metastasis and recurrence, requiring a wide and deep excision.

## Conclusions

The appearance of a subcutaneous mass may raise great concern regarding potential malignancy and uncertainty regarding prognosis. We elected to report this case due to its rare occurrence and the importance of identifying clear cell hidradenoma, a rare occurrence in cysts that can be successfully excised due to its benign nature. It is imperative to consider CCH among the differential diagnosis when evaluating and considering skin cysts, masses and tumors, as it may be difficult to differentiate it from other presentations. Additionally, through our research, we found that the presence of CCH in multiple lesions or recurrence of CCH should be monitored via long-term follow-ups to track potential malignant transformation to hidradenocarcinoma.

In our case, the incidental finding of an abdominal subcutaneous mass in a 60-year-old male led to suspicion for cancer or another malevolent underlying pathology. We were able to ascertain the benign nature of the mass and ruled out malignancy in this incidental finding via excision and pathological analysis. Through our findings, we hope to illustrate that although CCH is rare, it is important to include it as a potential diagnosis, due to the possibility of recurrence and malignant transformation, as well as its resemblance to malignant tumors despite its benign nature. Although this finding is rare, it depicts an excellent reason to send even benign-appearing lesions for pathologic examination.
